# Smart Microparticles with a pH-responsive Macropore for Targeted Oral Drug Delivery

**DOI:** 10.1038/s41598-017-03259-x

**Published:** 2017-06-08

**Authors:** Ankit Kumar, Carlo Montemagno, Hyo-Jick Choi

**Affiliations:** Department of Chemical and Materials Engineering, University of Alberta, and Ingenuity Lab, Edmonton, AB T6G 1H9 Canada

## Abstract

The development of a smart microencapsulation system programmed to actively respond to environmental pH change has long been recognized a key technology in pharmaceutical and food sciences. To this end, we developed hollow microparticles (MPs) with self-controlled macropores that respond to environmental pH change, using an Oil-in-Water emulsion technique, for oral drug delivery. We observed that freeze-drying of MPs induced closure of macropores. The closing/opening behavior of macropores was confirmed by exposing MPs encapsulating different ingredients (sulforhodamine b, fluorescent nanoparticles, and lactase) to simulated gastrointestinal (GI) fluids. MPs maintained their intact, closed pore structure in gastric pH, and subsequent exposure to intestinal pH resulted in pore opening and ingredients release. Further, MPs displayed higher protection (>15 times) than commercial lactase formulation, indicating the protective ability of the system against harsh GI conditions. This study showed development of a hybrid MP system combining the advantages of solid particles and hollow capsules, exhibiting easy solvent-free loading mechanism and smart protection/release of encapsulates through controllable macropores. Ultimately, our MPs system strives to usher a new research area in smart drug delivery systems and advance the current oral drug delivery technology by solving major challenges in targeted delivery of pH-sensitive therapeutics.

## Introduction

Environment-sensing microencapsulation systems have been extensively investigated due to their important capability to encapsulate biological/chemical reagents and therapeutic pharmaceuticals, and to subsequently release these agents in response to changes in environmental conditions^1–4^. Advancements in development of functional materials enabled researchers to exploit diverse stimuli to design and fabricate novel target-specific and time-controlled drug delivery systems. To this end, environmental stimuli (i.e., temperature, pH, ionic strength, mechanical strength, magnetic/electric fields, light, ultrasound, biomolecules, etc.) have been used to trigger a change in physicochemical properties of microencapsulation systems^5, 6^. For example, delivery system can be designed to swell or shrink in responses to temperature change-induced phase transition, electric field-induced polyion concentration change, pH-dependent protonation/deprotonation, and antigen-antibody interaction^7–13^. In addition, delivery systems can be equipped with on/off switches for drug release by applying magnetic field (i.e., magnetic heating), and light (i.e., photo-induced dissociation, photo-isomerization, photo-heating, generation of reactive oxygen species)^14–18^. Furthermore, cavitation of microbubbles triggered by ultrasound, volume phase transition by pressure, and redox-induced destabilization of delivery system have proven their potential as a stimulus-responsive delivery mechanism^19–27^. However, all environment-responding carriers must deal with the issues of (i) efficient loading and stability of the functional ingredients in the unfavorable environmental conditions, and (ii) the release control of the active reagents to exhibit full performance in response to targeted environmental conditions for desired effects^28^. Specifically, progress in currently investigated pH-responsive encapsulation systems for oral administration has been significantly restricted due to multiple technical limitations such as low encapsulation efficiency, incomplete protection of the encapsulated ingredients against harsh environmental conditions, inefficient response to stimuli, and incomplete release behavior. Therefore, considering the potential application of pH-responding oral drug delivery system in pharmaceutical science, it is important to evaluate technical challenges of the previously investigated systems and design new approaches to meet the demands of high-precision performance.

To date, microemulsion has been widely employed to fabricate microencapsulation systems for drug delivery (i.e., almost exclusively for solid particles and hollow capsules)^29–32^. The driving force for solid and hollow architecture is the advantage of providing convenient encapsulation of hydrophobic and hydrophilic drugs, respectively. In a typical preparation, hydrophobic drugs are mixed with a matrix forming material dissolved in organic solvent (oil phase: O), which is emulsified in aqueous solution (water phase: W), followed by organic solvent removal, harvest, and dehydration. For the encapsulation of hydrophilic drugs, single Oil-in-Water (O/W) and Water-in-Oil (W/O) emulsion techniques have been modified for efficient encapsulation of drugs. Alternative emulsion types investigated include O/O emulsion to reduce partitioning of drugs, W/O/W double emulsion, O/W emulsion by using cosolvents, and W/O/O double emulsion by selecting appropriate solvents for each emulsion type^29, 32, 33^. However, microencapsulation systems faced significant technical challenges in preserving full structural/functional stability during emulsion/particle formation and storage. This degradation of biopharmaceuticals is related to the fact that microemulsion and microspheres have been prepared from the same suspension in which drug has been dissolved for entrapment. That is, drugs are inevitably exposed to unfavorable environment such as organic solvent, water-oil interface in microemulsion, shear or cavitation stresses during agitation, and hydrophobic interaction with polymer matrix, resulting in denaturation and aggregation^34–38^. In addition, structural instability of biotherapeutics was associated with incomplete release profile in an uncontrollable manner, subsequently, restricting the application of current microencapsulation systems^39^. Thus, considerable efforts have been directed toward the modification of emulsion formulation or the adoption of different emulsification method to minimize exposure of biotherapeutics to destabilizing factors^37, 40–42^. As one would expect, a seemingly working condition cannot work equally well for other types of drugs/emulsion conditions, because of large variations in material properties and process conditions. Consequently, there has been a constant need to develop a target-specific, universally applicable microparticle (MP) system to eliminate destabilization of biotherapeutics during emulsification and MP preparation process.

The goal of this research is to develop an innovative oral drug delivery system which can meet multiple demands of convenient encapsulation, protection against MP fabrication and gastrointestinal (GI) environments, targeted delivery, and efficient release of functional ingredients simultaneously. For this purpose, we report a smart microencapsulation system with self-controlled macropores that respond to environmental pH change (see Fig. S1, Supporting Information). Our strategy is to first fabricate MPs with pH-responding macropores using microemulsion technology, and then encapsulate drugs into MPs through their pores. Since MP fabrication and drug encapsulation occur through independent processes, the critical issue of above-mentioned destabilization of biopharmaceuticals in the conventional microemulsion-based drug delivery systems can be resolved by adopting the proposed architecture, i.e. MP with a macropore. In particular, the presence of a macropore will provide unique advantages such as easy loading of biopharmaceuticals with high efficiency, a promising platform for wide size range of active drugs such as DNAs, peptides, antibodies, enzymes, proteins, nanoparticles (ranging from a few nanometers to hundreds of nanometers), and whole inactivated virus vaccines (~100 nm). The key structural feature of our system is the presence of pH-responsive macropores which sense and adapt to environmental pH changes (Supplementary Fig. S1a). This means that functional substances encapsulated in these delivery systems would be protected in acidic pH environment of stomach due to the closure of the pores (Supplementary Fig. S1b, c), and would be rapidly released in the targeted pH environment of intestine due to the opening of the macropores (Supplementary Fig. S1d).

To achieve the aforementioned objectives, this study presents development of the concepted smart pH-responding pored microencapsulation system using microemulsion technology via sonication. Following the design and fabrication of pored MPs, their performance in the digestive pH conditions was further investigated to evaluate the feasibility of our novel pH-sensing oral drug delivery system. Hence, the initial focus of our research was to optimize emulsion forming conditions and process variables for the fabrication of pored MPs, followed by freeze-drying process to seal the pores. pH-responsive pore closure/opening, and loading/release behavior of model drugs (sulforhodamine b and 100 nm size fluorescent polystyrene nanoparticles) were tested in simulated GI tract conditions by monitoring morphological change of MPs through electron microscopy and fluorescence studies, respectively. Later, lactase, as a model pH-sensitive functional ingredient, was encapsulated into MPs, and its functional activity was assayed using ortho-nitrophenyl-β-galactoside (ONPG) to confirm the preservation of enzymes in the simulated gastric environment via closed pored MP formulation, and to ensure release of the enzyme in the simulated intestine environment through pore opening.

## Methods

### Fabrication of MPs with a macropore

A new and simple method was developed to make MPs with macropores using modified O/W emulsion solvent evaporation method. Eudragit S100 polymer used for MPs preparation was received as a generous gift from Evonik Canada Inc. (Burlington, ON, Canada). Aqueous phase emulsifier-poly (vinyl alcohol) (PVA), Mw: 9,000–10,000, and detergent-Tween 20, Fisher BioReagents, were purchased from Sigma-Aldrich (St Louis, MO, USA) and Fisher Scientific (Missisauga, ON, USA), respectively. 5 wt% Eudragit S100 polymer is dissolved in the cosolvent system (dichloromethane:ethanol:isopropanol = 2:1:1) to prepare oil phase, 10 mL of which is added into a 190 mL of aqueous phase containing 0.5% PVA and 5% Tween 20 to make O/W emulsion by applying sonication at 30 W for 30 min (GE-130 ultrasonic processor, Sonics & Materials, Inc.; Newtown, CT). Post sonication, emulsion was stirred at room temperature (R.T.) for 10 min, followed by another sonication cycle for 30 min. The emulsion was centrifuge-washed four times with DI water at 12,000 g for 20 min (Eppendorf Model 5810; Hamburg, Germany). The concentrated residue suspension was rotary evaporated to remove remaining solvents at 65 °C for 5–10 min (Büchi R200; Labortechnik, Flawil, Switzerland). Afterwards, the final suspension was vacuum filtered using Whatman filter papers (grade 4) to collect MPs (<20 μm). Filtered samples were concentrated and freeze-dried for further analysis.

### Study of different sonication methods on emulsion temperature and MPs properties

To control the evaporation rate of solvent from MPs, multiple process conditions were tested including sonication time and total sample volume (50, 100, and 200 mL; 5 v/v% of polymer stock solution). Specifically, the sample temperature was measured after a continuous sonication (sonication cycle on throughout) at R.T. (i.e., no external temperature control) and in iced water for varying total sonication time durations (5, 10, 15, 30, and 60 min). Morphologies of MPs prepared by sonicating 100 mL of samples for 5, 30, and 60 min were analyzed by scanning electron microscope (SEM; S4800 Electron Microscope; Hitachi, Japan) to investigate the effect of sample temperature on the pore forming properties of MPs.

### Controllability of pore characteristics by slow solvent evaporation of MPs

In case of R.T. stir incubated MPs, 5 wt% Eudragit S100 polymer is dissolved in the co-solvent system (Dichloromethane:Ethanol:Isopropanol = 2:1:1) and added into an aqueous phase of DI water containing 0.5% PVA and 5% Tween 20 to make O/W emulsion by applying sonication (30 W, 5 min) at R.T. For the iced water stir incubated MPs, the emulsion is prepared in a similar manner but the sonication is applied under iced water condition (container was surrounded by iced water). In both cases, sonicated emulsion was further magnetically stirred (700 rpm) at R.T. for a specified time interval (0, 2, 4, and 8 hrs), followed by centrifuge washing (12,000 g, 20 min) and rotary evaporation (65 °C, 5–10 min). Samples were filtered, concentrated, and freeze-dried as previously described for further characterization.

### Pore closure by freeze-drying

To close pores of MPs, a new method was developed using freeze-dryer. Initially, concentrated MPs suspension in water was stored in a 2 mL Eppendorf tube and then frozen using liquid nitrogen. The frozen sample was then transferred to freeze-dryer (pre-cooled to −40 °C) to undergo freeze-drying process using an in-house recipe, built considering polymer properties and cosolvent system as shown in Fig. S2 (AdVantage Pro Freeze Dryer, SP Scientific; Warminster, PA). Samples were stored in 4 °C refrigerator after collection for further use.

### pH responsiveness of MPs

To test pH-responsive behavior of MPs, samples were subjected to two kinds of buffers – acidic and neutral. Acidic buffer having a pH 2.0 was prepared using a potassium chloride (KCl)/hydrochloric acid (HCl) buffer. In a typical method, 0.1 wt% KCl solution was kept on a magnetic stirrer plate, and then 0.1 wt% HCl was added dropwise to adjust the final pH to be 2.0. To prepare neutral pH buffers, 0.1 wt% disodium phosphate (Na_2_HPO_4_) was added dropwise to KCl/HCl acidic buffer until the final pH reached to 7.1. Lyophilized MPs powder was suspended in prewarmed acidic buffer, and further incubated for 2 hrs at 37 °C in oven to simulate gastric environment. Half of the sample was collected for further characterization, and the rest half was mixed with 0.1 wt% disodium phosphate (prewarmed to 37 °C) to make the final pH 7.1, which corresponds to the intestine pH. It was incubated for 4 hrs to simulate intestine digestion time.

To further demonstrate the pH-responsiveness of pored MPs system, MPs were exposed to modified simulated gastric and intestinal fluid with enzymes for 2 hrs and 4 hrs, respectively. The gastrointestinal fluid was prepared following U.S. Pharmacopeia (USP) guideline with the modification of the final pH in this study. For simulated gastric fluid with enzyme, 0.2 wt% of NaCl and 0.32 wt% of pepsin (derived from porcine gastric mucosa; Sigma-Aldrich) were dissolved in DI water, and the pH was adjusted to 2.0 by drop-wise adding 0.2 M HCl to the solution. To prepare simulated intestinal fluid with enzyme, 0.68 wt% of monobasic potassium phosphate and 1 wt% pancreatin (from porcine pancreas; Sigma-Aldrich) were dissolved in DI water, and 0.2 M NaOH or 0.2 M HCl was added to the solution to adjust the final pH to 7.1. Similar protocol was followed as described above to study pH-dependent pore closing/opening behavior of MPs.

### Sulforhodamine b (SRB)/fluorescent nanoparticle (FNP)/lactase encapsulation

50 mg of freeze-dried MPs for SRB and FNP, and 300 mg MPs for lactase encapsulation were transferred to a 2 mL and 50 mL eppendorf tube, respectively. MPs were suspended in 300 μL of 300 mM trehalose solution in DI water with 0.25 mM SRB (Sigma-Aldrich). Similarly, in case of FNP (FluoSpheres® carboxylate microspheres, 0.1 µm; Thermo Fisher Scientific), MPs were suspended in 1 mL nanoparticles solution diluted five times in 300 mM trehalose solution. Likewise, in case of lactase (β-galactosidase from Aspergillus oryzae; Sigma-Aldrich), MPs were suspended in 12 mL of 20 mg·mL^−1^ lactase solution containing 15% trehalose and 0.5% carboxymethyl cellulose (Sigma-Aldrich)^43^. All samples were transferred into vacuum oven with vacuum on/off cycle for 4 to 5 times. This ensured that all the air pockets were removed from pored MPs and were replaced by ingredients (i.e. SRB, FNP, and lactase). Non-encapsulated SRB/FNP/lactase were separated by centrifugation at 10,000 rpm for 10 min. SRB-/FNP-/lactase-MP pellets were resuspended in DI water by brief vortexing for 1–2 s, and plunged into liquid nitrogen. All frozen samples were freeze-dried by employing the same recipe as described in Table S1.

### SRB/FNP/lactase release

A suspension of freeze-dried sample in pH 2.0 buffer (300 μL for SRB and FNP, and 1 mL for lactase) was centrifuge-washed twice to ensure complete removal of the non-encapsulated ingredients at 10,000 rpm for 10 min. Then sample was incubated for 2 hrs at 37 °C in oven to simulate gastric environment. Post incubation, sample was mixed with disodium phosphate (prewarmed to 37 °C) to make the final pH 7.1 and incubated for an additional 4 hrs to simulate intestine environment.

### Electron microscopy analysis

SEM and transmission electron microscope (TEM; JEOL JEM 2100; JEOL, Peabody, MA) were utilized to observe the morphology of fabricated MPs, pH responsiveness, and release experiments, and for confirming the closing and opening of MPs. For SEM analysis, MPs suspension was placed on a glass coverslip which was fixed to a double-sided carbon tape attached to an aluminum stub, and dried overnight at R.T. A 5-nm thick gold layer was deposited on the samples to minimize the charging effect prior to observation at 15 kV (10 μA). In the case of TEM, 7 µL of MP suspension was placed on the formvar-carbon-coated copper grids (Electron Microscopy Sciences, Hatfield, PA), blotted with a filter paper after 7 min, and the grids were dried at ambient conditions prior to TEM analysis (200 kV).

### Fluorescence measurement

Fluorescence of released model drugs (FNP and SRB) were measured on a Flexstation 3 benchtop multi-mode microplate reader (Molecular Devices; Sunnyvale, California, USA). Fluorescence spectra for FNPs were monitored at the excitation of 490 nm and emission range of 500–530 nm, with a 1 nm step size. For SRB experiments, excitation wavelength and emission range were fixed at 540 nm and 565–595 nm, respectively. All the measurements were made in Corning 96 wells clear bottom plate with 200 µL sample volume in individual well. Cover tape was used between each experiment to prevent the evaporation of sample that takes place due to long incubation time.

### Fluorescence microscopy

Fluorescence microscopy analysis was performed using an Olympus IX81 inverted microscope (Olympus, Germany) coupled with DP 80 digital camera having dual CCD sensors. CellSens software (Olympus, Germany) was employed to obtain micrographs captured at 40× objective (Olympus LCPlanFl, 1 µm depth of field, NA 0.6). FITC and TRITC filters were used for FNP and SRB fluorophores, respectively. Samples were placed on a glass slide and imaged after covering with a glass cover slip.

### Lactase activity test

A modified colorimetric assay from a reported protocol was utilized to determine lactase activity in which ONPG undergoes enzymatic cleavage, resulting in formation of yellow color o-nitrophenolate^44^. In a 96-well plate, 50 μL of solution containing released lactase from MPs post pores opening in pH 7.1 buffer was mixed with 5 μL working buffer (50 mM MgSO_4_/20 mM HEPES/NaOH pH 7.2) and 100 μL ONPG solution (14 mM in DI water, Sigma-Aldrich). The mixture was incubated for 2 hrs at 37 °C and measured for absorption values at 415 nm. To calculate the survival rate of lactase encapsulated in MPs, functional activities were compared with original lactase in pH 2.0, pH 7.1, and in pH 2.0 followed by pH 7.1 solutions using the intact lactase in pH 7.1 buffer as reference standards. For testing the survival rate for commercial lactase sold as dietary supplement, enzyme from same source Aspergillus oryzae was purchased. Briefly, each tablet was dissolved in 200 mL of pH 2.0 and 7.1 buffer solutions, which were incubated for 2 and 4 hrs, respectively, to simulate GI tract environment. After incubation, 50 μL of solution from each suspension was taken out and subjected to ONPG assay described above. A standard from commercial lactase suspension in pH 7.1 buffer was used as reference standards.

### Fourier transform infrared (FTIR) spectroscopy

To analyze the residues of PVA and Tween 20 in the MPs, FTS 7000 FTIR spectrometer (Varian Inc., Palo Alto, CA, USA) equipped with deuterated triglycine sulfate (DTGS) detector, was used to measure the absorbance spectra with a spectral resolution of 4 cm^−1^. Spectra for freeze-dried MPs were compared with the individual spectra of PVA, Tween 20 and original polymer.

### Nuclear magnetic resonance (NMR) spectroscopy

To confirm the results obtained from FTIR analysis, NMR spectroscopy was performed with Varian Direct Drive VNMRS 600 spectrometer (Agilent Technologies, Santa Clara CA) operating at a magnetic field strength of 14.1 T (600 MHz ^1^H frequency) using dimethyl sulfoxide-d_6_ (DMSO-d_6_) solvent. Measurements were acquired at R.T. using a single pulse excitation with a 45° flip angle of 3.6 µs. Acquisition time was maintained at 1.7 s with a repetition time of one second. Polymer, PVA and Tween 20 spectra were recorded separately and used as references for comparison with MPs’ spectra.

### Differential Scanning Calorimetry (DSC)

To observe any changes in the glass transition temperature (T_g_) of MPs, DSC measurements were carried out with a Pyris-1 DSC (Perkin-Elmer, Norwalk, CT, USA) at heating and cooling rates of 10 °C·min^−1^ and at a scan rate of 1 °C·min^−1^. The samples were first cooled from R.T. to −150 °C, and then heated to 200 °C following 1–2 hr of equilibration in the instrument. For comparison purposes, T_g_ of original polymer powder was measured.

### Statistics

Data were analyzed using student’s t-test in Minitab software (State College, PA, USA). A *P*-value of less than 0.05 indicated a significant difference.

## Results

### Fabrication of MPs with a pH-responsive macropore

To satisfy both pH stability in stomach and rapid release in intestine, we designed the MPs using anionic copolymers, whose pH-responsive behavior can be used to control pore closure and opening for protection and release of therapeutic agents in low pH and high pH, respectively. In this work, Eudragit S100, a copolymer composed of methacrylic acid-methyl methacrylate copolymer (1:2 ratio of MAA: MMA, Mw = 125 kDa), was used to synthesize MPs. This PMAA-PMMA copolymer has been extensively used in FDA approved enteric formulations and is known to dissolve at specific pH (≥7.0) due to the ionization of carboxylic acid groups^45, 46^. For this reason, pored MPs made of PMAA-PMMA copolymer can have unique ability to respond to pH change of gastric to intestinal environment, thus satisfying pH stability in acidic environment of stomach and triggered release in near neutral pH environment of intestine.

To make hollow MPs with macropores, our initial efforts were primarily focused to devise a sonication method with an optimal emulsion formulation by controlling evaporation of solvents. Briefly, polymer stock solution in the cosolvent (i.e., a mixture of dichloromethane, ethanol, and isopropanol) is added into an aqueous solution (0.5% PVA, 5% Tween 20) to make O/W emulsion by applying sonication. After stirring at R.T. for 10 min, the mixture was sonicated for an additional 30 min. This is a simple process to fabricate hollow MPs with a macropore, and the overall process is less time consuming (few hours), which does not require long incubation in vacuum conditions like the method used for polystyrene MPs^47^. Notably, this process showed excellent repeatability of forming pored MPs even though it employs co-solvent system, which was hard to achieve from previously reported method due to the difficulty in controlling solvent evaporation.

**Figure 1:**
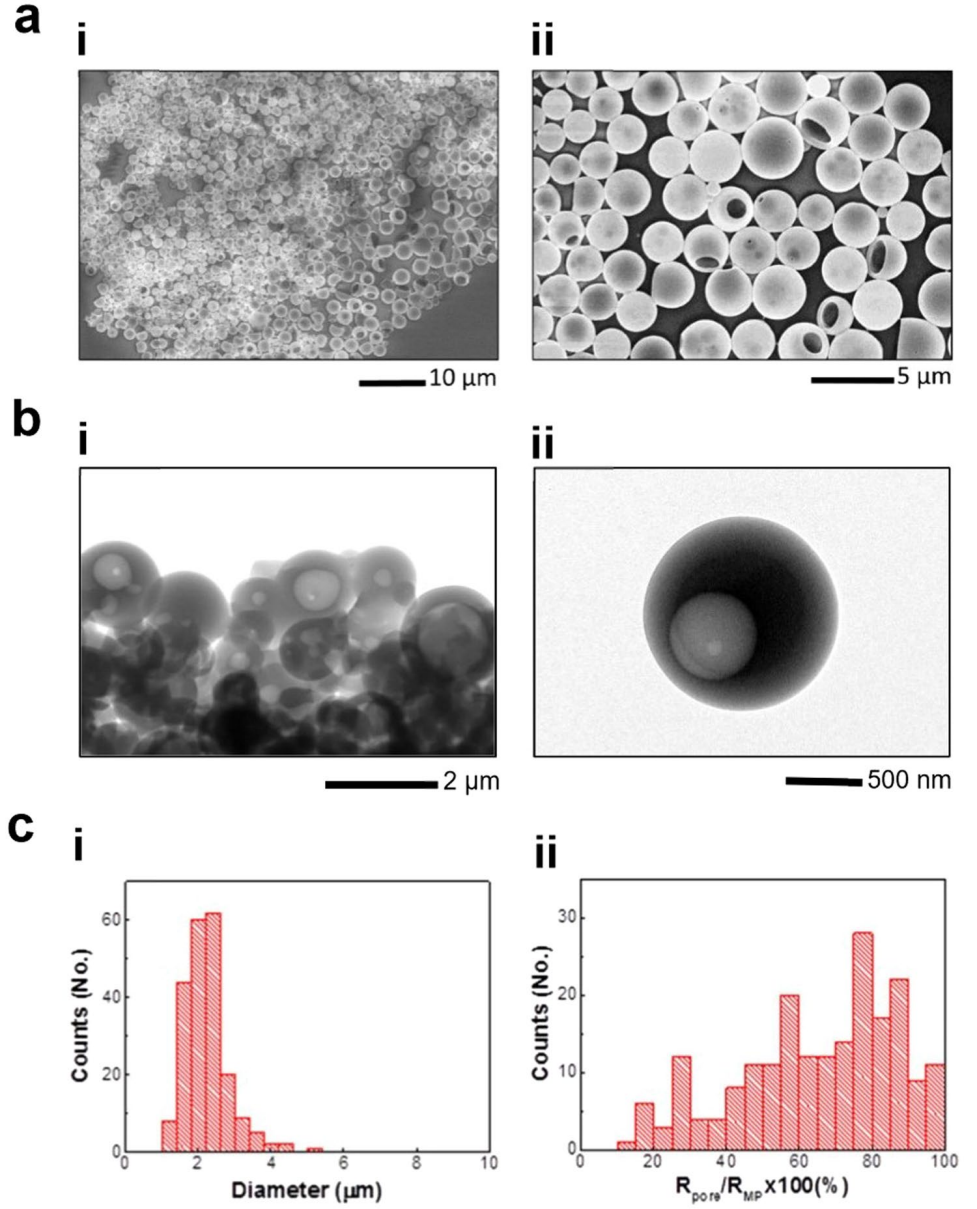
Fabrication and size distribution of MPs with a macropore. (**a**) SEM and (**b**) TEM micrographs of the fabricated MPs (i: low mag. and ii: high mag.) Pored MPs were successfully fabricated using pH sensitive polymer, Eudragit S100 by a newly developed method. (**c**) MP size distribution (i) and pore to MP size ratio (radius of pore (R_pore_)/radius of MP (R_MP_)) distribution (ii) obtained from SEM images of pored MPs (*n* = 210).

As shown in Fig. 1a and b (i: low mag. and ii: high mag.), SEM analysis revealed the successful formation of spherical hollow MPs with a macropore, which is further supported by TEM observation (see Fig. 1a for SEM and Fig. 1b for TEM images). From the SEM image analysis of 210 pored MPs, the average diameters of MPs and their pores were measured to be 2.2 ± 0.6 µm and 1.5 ± 0.7 µm, respectively (Fig. 1c(i)). This corresponds to 66.4 ± 22.4% of pore-to-MP size ratio (see Fig. 1c(ii) for distribution histogram). The successful formation of a macropore can enable efficient solvent-free loading and delivery of various therapeutic substances ranging in size from few nanometers to hundreds of nanometers.

### Closing and opening of the macropore in response to pH

**Figure 2:**
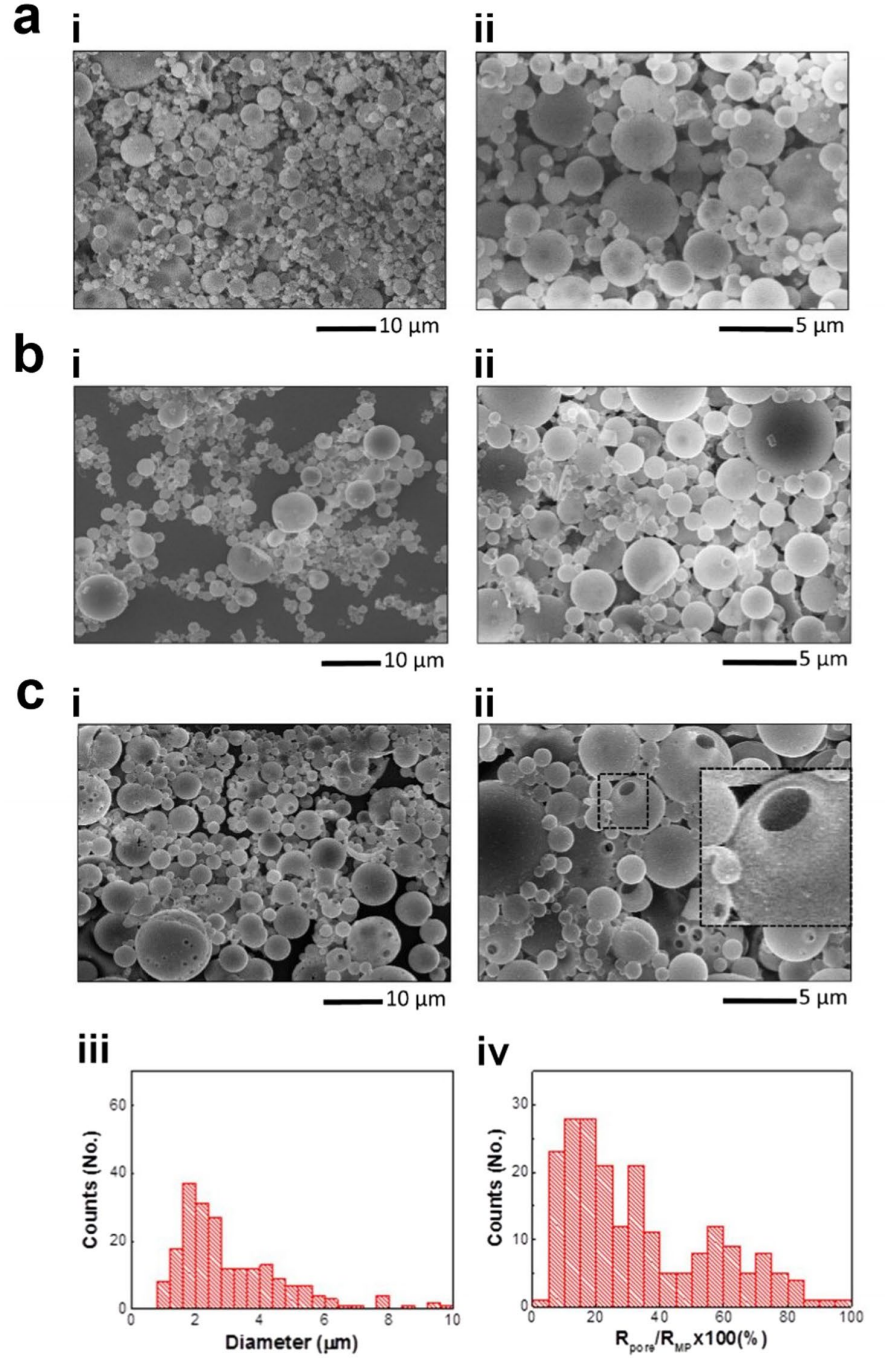
Pore closure and opening in response to pH. SEM images of MPs after pore closure by freeze-drying (**a**), and after being subjected to 2-hr incubation at pH 2.0 and 37 °C (**b**) (i: low mag. and ii: high mag.) (**c**) SEM images of MPs after being subjected to simulated GI tract conditions (2-hr incubation at pH 2.0, followed by 4-hr incubation at pH 7.1 at 37 °C, i: low mag. and ii: high mag.), i.e. after 4-hr incubation of the (**b**) sample at pH 7.1 and 37 °C (inset: × 3), and size distribution of MPs (iii) and Pore-to-MP size ratio (iv) analyzed from SEM images of MPs as shown in **c** (i, ii**)** (*n* = 210).

Following the fabrication of MPs with a macropore, one of the most significant technical challenges was to develop a method to completely seal the pores of MPs. While pores are open to allow for easy loading, they must be closed to preserve functional activity of materials against acidic pH of stomach. At the same time, the pore sealing method must be compatible with existing pharmaceutical manufacturing process. For this purpose, we found an efficient way of closing the pores of MPs by freeze-dry process (see **Methods** and Fig. S2, Supporting Information). As shown in Fig. 2a, it is clear that freeze-drying caused pore closure as analyzed by SEM (see Fig. 1a for the original pored MPs and Fig. 2a for MPs after the application of freeze-drying). Since this method does not require annealing at high temperature nor exposure to organic solvents, it can be universally applicable in sealing the pores of MPs without destabilizing encapsulated pharmaceuticals.

After the sealing of MP pores, time-dependent pH response of macropores was investigated in the simulated GI tract environment. Closed-pored MPs were subjected to pH 2.0 buffer at 37 °C for 2 hrs and then subsequently to pH 7.1 buffer at 37 °C for 4 hrs to study their behavior in stomach and intestine environment, respectively. SEM analysis confirmed that the pores of the MPs remain closed by the exposure and incubation in pH 2.0 buffer, displaying no sign of structural change in polymer and/or MPs (compare Fig. 2b with 2a). This is consistent with our TEM analysis as shown in Fig. S3a (Supporting Information). Considering that major destabilization of pharmaceuticals occurs in acidic environment of stomach, the maintenance of a completely closed-pore state at acidic pH is hypothesized to provide full protection against low pH of stomach. However, when the pH was controlled to be at 7.1, MPs reopened their pores, as can be seen clearly from the SEM and TEM images (Fig. 2c(i) and (ii) for SEM, and Fig. S3b in Supporting information for TEM), which can be explained by the pH-responsive properties of Eudragit S100 polymer. These data support the pH-responsive behavior of the macropore of MPs.

MPs with reopened pores exhibited more polydisperse size distributions of both MPs and their pores (MP diameter: 3.1 ± 1.7 µm, pore diameter: 0.85 ± 0.55 µm) than those of open-pored MPs before freeze-drying (see Fig. 2c(iii) and (iv) for particle diameter and pore-to-MP size ratio, respectively). It is also noted that in contrast to MPs with open pores, MP population showed an increasing trend with decreasing pore-to-MP size ratio. As a result, MPs with reopened pores exhibited a lower ratio of pore-to-MP size, 33.3 ± 22.6%, compared to that of open-pored MPs (Student’s t-test, *P* < 0.001; see Figs 1c(ii) and 2c(iv)). Despite the difference in the pore-to-MP size ratio between original pored MPs and reopened-pored MPs, it is reasonable to assume that the MPs maintains their intact structure in acidic gastric environment, but swells and/or dissolves in neutral intestinal environment to reopen their pores.

### Encapsulation of model drugs and study of their release behavior

**Figure 3:**
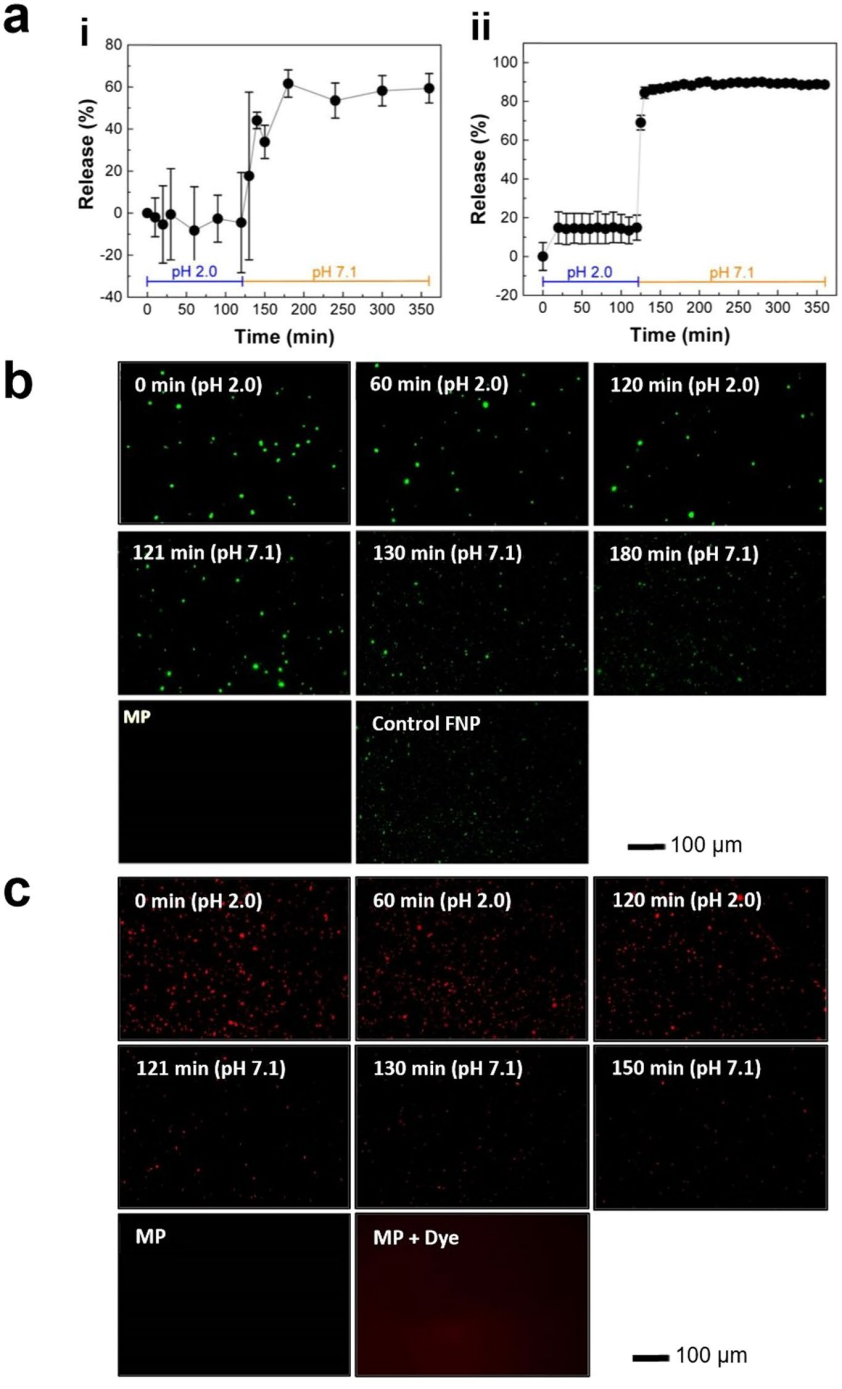
Release of ingredients from MPs in digestive conditions. (**a**) Time dependent release profile of encapsulated 100 nm FNPs (i) and SRB (ii) from MPs subjected to simulated GI tract environment (stomach: 2-hr incubation at pH 2.0 and 37 °C, and intestine: 4-hr incubation at pH 7.1 and 37 °C). (**b**), (**c**) Representative fluorescence micrographs of (**b**) FNP- and (**c**) SRB-encapsulating MPs after incubation at simulated gastric and intestinal conditions. As a control, fluorescent micrographs of MPs without FNP and SRB are shown for comparison.

To confirm the hollow interior of MPs as predicted from SEM analysis, and to test potential applicability of pored MPs for targeted delivery of various ingredients to a GI tract, two model drug-encapsulated MPs with closed pores were prepared, i.e. fluorescent polystyrene nanoparticles (FNPs; 100 nm) and sulforhodamine b (SRB; 580 Da). Fluorescence measurements were performed for FNP- and SRB-encapsulated MPs (hereafter abbreviated as FNP-MPs and SRB-MPs, respectively) to monitor their encapsulation and pH-dependent release behavior through pH-responsive pores. To this end, the variation in fluorescence intensity was measured over the increase of incubation time, i.e. 2 hrs at pH 2.0 and 4 hrs at pH 7.1. As shown in Fig. 3a(i), no significant level of fluorescence intensity change was observed during the 2-hr incubation period at pH 2.0. This is associated with the absence of FNP leakage from MPs due to closed pores. However, exposure to pH 7.1 induced a significant FNP release during the initial 20 min, followed by saturation in release profile around 60–65% within an hour. This release can be explained by pH-induced opening of MP pores, as predicted from SEM analysis in Fig. 2c. On the other hand, as shown in Fig. 3a(ii), SRB-MPs exhibited a biphasic release behavior: an initial release up to 14–20% at 20 min upon exposure to pH 2.0, followed by a rapid increase up to 85–90% within 10 min. As indicated in the plateau at 20 min, the absence of further fluorescence intensity change indicates that the leak occurring at the beginning of sample incubation could be attributed to SRB from remaining MPs with incomplete sealed pores. When exposed to pH 7.1, a steep increase in release rate was followed by a plateau (85–90%) within 10 min. The more rapid release profile of SRB-MPs can be explained by the smaller size of SRB compared to FNPs. This pH-responsive behavior of FNP-/SRB-MPs is consistent with our pH-dependent morphological change of MPs as shown in Fig. 2. It is important to note that time-dependent release tests shown in Fig. 3a have been performed without application of any mechanical agitation, purely to monitor release behavior of ingredients due to pH-responsive closing/opening of pores. However, a brief mechanical agitation by vortexing confirmed complete release of FNPs and SRB from MPs.

This time- and pH-dependent release behavior of MP contents is further supported by fluorescence microscopy analysis. As can be seen in the Fig. 3b and c, FNP- and SRB-MPs maintained their intact structure over the course of incubation at pH 2.0 for 2 hrs, thus no significant difference was observed due to leakage of ingredients. However, when exposed to pH 7.1, MPs showed a time-dependent leakage for both FNPs and SRB, which can be explained by pH-triggered pore opening. These experiments indicate that the MPs with pH-responsive macropores can encapsulate model drugs and protect them from the gastric environment, and release them in intestine environment.

### Preservation and delivery of lactase to the intestine

**Figure 4:**
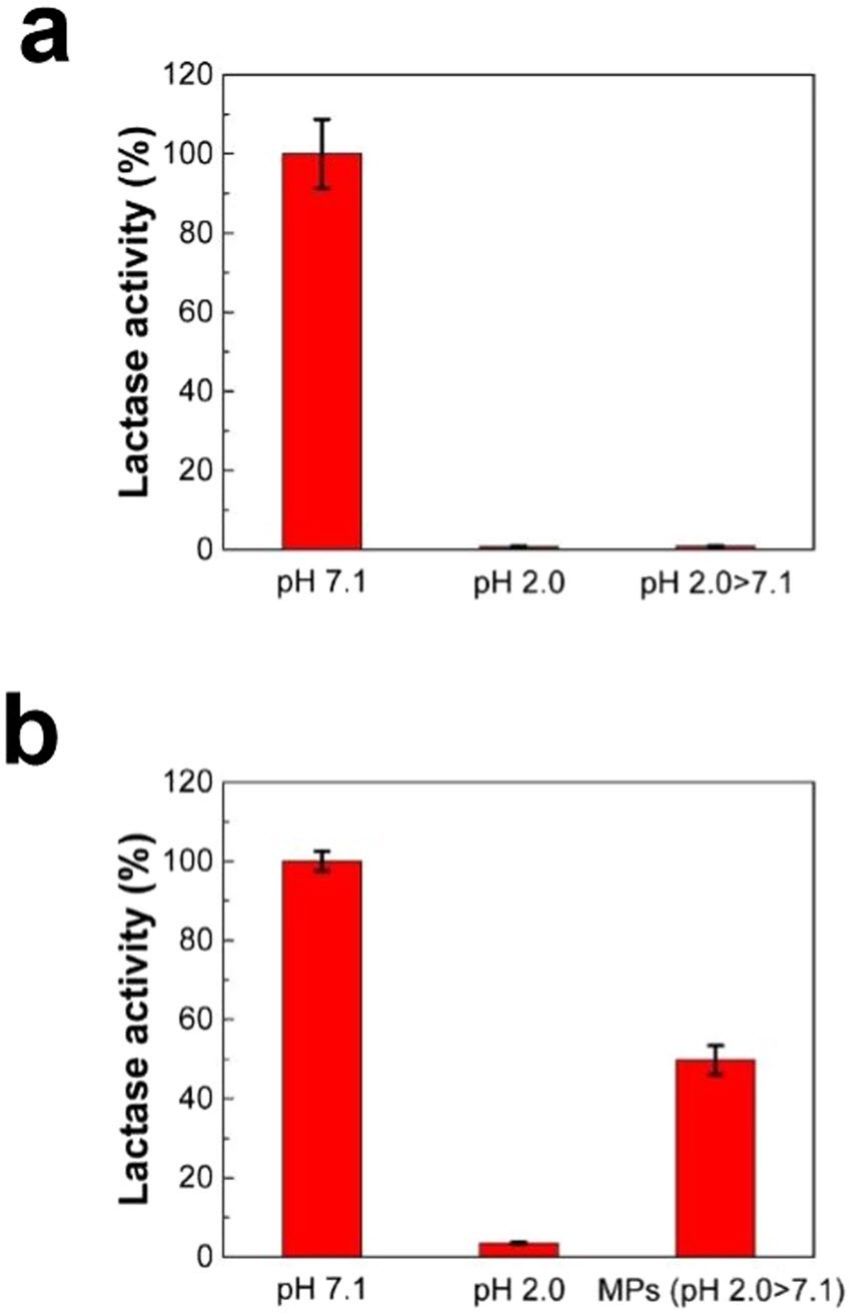
Protective effect of MP formulation on the functional activity of lactase. The relative activities of (**a**) a commercial lactase tablet and (**b**) MP-encapsulated lactase in simulated GI tract conditions (*n* = 6, mean ± SD). Functional activity of lactase was calculated relative to that of control lactase at pH 7.1. In (**a**) and (**b**), pH 7.1 and 2.0 represent activity of lactase measured at pH 7.1 and 2.0, respectively, without using MPs. pH 2.0 > 7.1 represents activity of lactase in simulated GI tract conditions.

After the proof-of-concept experiments for the model drug-encapsulating MPs, we examined the potential applicability of MPs as lactase carriers for alleviating lactose intolerance^48–51^. This is based on the hypothesis that protection of lactase due to pore closure at acidic pH of stomach followed by release at neutral pH of intestine would maintain their initial functional activity and provide a dose-sparing effect. To compare relative efficacy of lactase formulations, ONPG assay was performed for commercial lactase tablets and lactase-encapsulating MPs at different pH conditions: positive control, pH 7.1 (4-hr incubation at pH 7.1); negative control, pH 2.0 (2-hr incubation at pH 2.0); GI tract, pH 2.0 > 7.1 (2-hr incubation at pH 2.0, followed by 4-hr incubation at pH 7.1)^44^. As shown in Fig. 4a, commercial tablet exhibited a significant loss of lactase activity at pH 2.0 (remaining activity: 0.7 ± 0.3%), indicating that acidic condition of stomach is a critical factor in destabilizing lactase. As such, no significant level of remaining activity was observed under simulated GI tract conditions (remaining activity of pH 2.0 > 7.1: 2.9 ± 0.6%). Similarly, the activity of the lactase used for MP preparation was significantly reduced at pH 2.0 (Fig. 4b). However, it is notable that about 50% of functional activity was preserved in the case of lactase-MP formulation. This significant increase in remaining activity is associated with improved protection of lactase enzyme against unfavorable acidic pH condition due to the pH-responsive MPs, consistent with our previous data in Figs 2 and 3. Since protective activity of MPs with pH-responding pores is associated with the extent of pore closure, we expect that the pore closure efficiency would be an important factor in determining the remaining activity of lactase. Therefore, although further research can improve pore closure efficiency, our results support that pH-responding smart microencapsulation system can counteract the technical challenges associated with the need of administration of high dose lactase due to its destabilization in the stomach.

## Discussion

In efforts to develop a universal platform for environment-sensing microencapsulation system, first-of-its-kind MPs with pH-responsive pores were synthesized and their potential was investigated in this work. To implement proof-of-concept oral drug delivery system, we initially adopted the freeze-drying method, which has been used for polystyrene, to synthesize polystyrene based microcapsules with macropores on their surface^47^. Briefly, a microparticle emulsion is frozen in liquid nitrogen, followed by a slow evaporation of the solvent at <0 °C. In this technique, the critical ideas are to control the evaporation rate of the solvent and that the melting temperature of the solvent should be below 0 °C. While the idea has proven to be useful in simple material/solvent system such as polystyrene in chloroform, we found that it is extremely difficult to extend the technique to the Eudragit S100, probably due to the prerequisite of the complicated cosolvent system as the organic phase in O/W emulsion. Specifically, the use of cosolvent mixtures with different melting (T_m_) and boiling (T_b_) temperatures, i.e. dichloromethane (T_m_: −96.7 °C, T_b_: 39.6 °C), ethanol (T_m_: −114 °C, T_b_: 78.4 °C), isopropanol (T_m_: −89 °C, T_b_: 82.5 °C), may have increased the difficulty in controlling both evaporation rate and emulsion stability, parameters critical to the formation of MPs and pores.

To solve these issues, a great deal of attention has been paid to the emulsion forming conditions and solvent evaporation conditions during the sonication process: sonication time, emulsion temperature, and sample volume. This is reasonable because pore formation of MPs would strongly depend on the evaporation of organic solvents due to the generation of heat during sonication process. Especially, the co-solvent system used in this work to make organic phase in the O/W emulsion is mainly composed of dichloromethane which has relatively low boiling temperature (T_b_: 39.6 °C).

Considering the hypothesis that evaporation rate of dichloromethane directly affects the size of pores, we performed experiments to measure the temperature of the samples over increasing sonication times (5, 10, 15, 30, and 60 min) for three different sample volumes (50, 100, and 200 mL) containing 5 v/v% of organic phase and 95 v/v% of aqueous phase (0.5% PVA/5% Tween 20). In addition, to test potential controllability of sample temperature during sonication, emulsions were prepared under two different environments: sonication at R.T. (i.e., no temp. control) and in iced water (i.e., temperature of sample being controlled by iced water during sonication).

**Figure 5:**
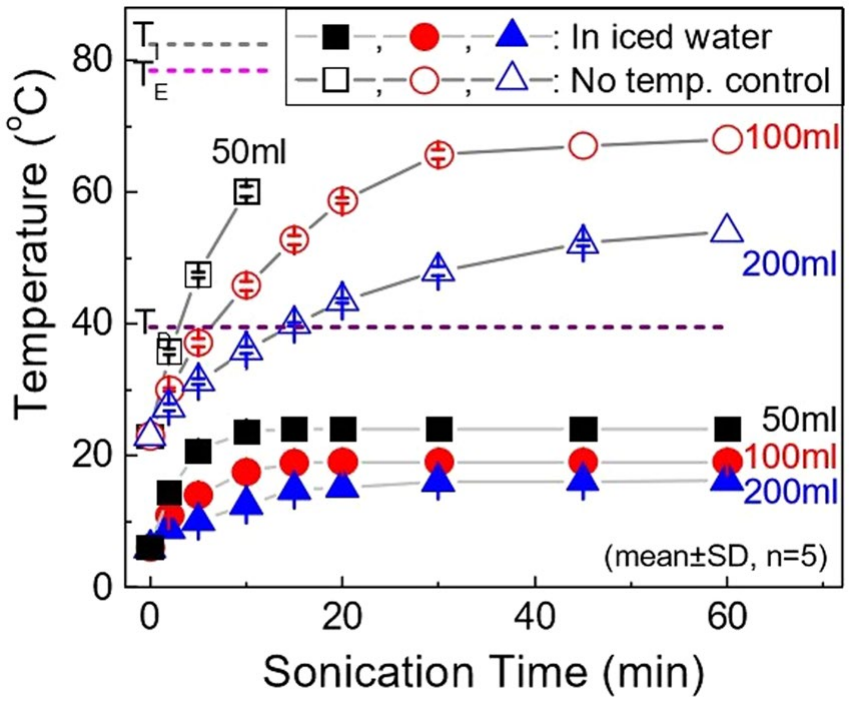
Temperature profiles of emulsion prepared with different sonication conditions. Three different sample volumes were emulsified with different sonication times (5, 10, 15, 30, and 60 min) by sonication at 30 W for temperature monitoring: no temp. control (sonication at room temperature, i.e. R.T. sonication) and in iced water (temperature of sample being controlled by iced water during sonication, i.e. iced water sonication) (*n* = 5, mean ± SD).

As shown in the plot of Fig. 5, sample temperature increased with longer sonication time, and decreased with larger sample volume for continuous sonication at R.T. The temperature of the room-temperature sonicated 50 mL-sample rose close to 60 °C within 10 min, making it extremely difficult to control to evaporation rate of solvents. In the case of 200 mL sample volume, although the sample temperature rose in a controlled manner, the large volume made it difficult to form stable emulsion for different sonication conditions. On the other hand, for the 100 mL sample, the temperature rose close to boiling point of dichloromethane in 5 min, followed by a gradual increase up to 68 °C in a controlled manner with the increase of sonication time. In addition, 100 mL sample size made the most stable microemulsion under our sonication conditions.

In the case of sonication in iced water, samples exhibited a slow increase in temperature over the increase of sonication time. On the contrary to R.T. sonication, sonication in iced water did not show any significant temperature increase. The cooling of the sample container would reduce the temperature rise that is caused by heat generated during sonication. As a result, for all the different sample volumes, the temperature remained below the boiling point of dichloromethane. Since rapid evaporation would make it difficult to control the size of MP pores, cooling the sample by iced water would provide better control on the evaporation rate of solvent.

To study the effects of sonication conditions on MP morphology, SEM analysis was performed for both non-controlled (R.T. sonication) and controlled environment (iced water sonication) samples prepared by sonicating 100 mL of samples at three different time intervals (5 min, 30 min, and 60 min) (see Supplementary Figs S4 and S5 for R.T. and iced water sonicated samples, respectively). As shown in Fig. S4a, 5 min R.T. sonication did not exhibit spherical morphology, neither pore formation on their surface. This can be explained by the formation of unstable emulsion and negligible solvent evaporation due to short duration sonication and incubation. On the other hand, as expected from the temperature increase >60 °C in Fig. 5, both 30 and 60 min sonication duration produced pored MPs with a macropore, supporting our hypothesis regarding the important role of sample temperature in pored MP formation. This indicates that continuous sonication for 5, 10, and 20 min can ensure the formation of pores for 50, 100, and 200 mL of samples, respectively, because sample temperature would reach T_b_ of dichloromethane. In addition, the observation of heterogeneous pore size distribution can be explained by rapid solvent evaporation. Therefore, although the MPs prepared by R.T. sonication maintained their spherical morphology, less control was observed in terms of the pore formation. In contrast, MPs prepared by iced water sonication required more than 30 min to form MPs with spherical morphology (Fig. S5). Moreover, iced water sonicated MPs appeared to form smaller pores in comparison with R.T. sonicated MPs, which can be explained by slower solvent evaporation rate. Hence, these results supported the possibility of controlling the pore size of MPs by sample temperature, and the importance of slow solvent evaporation.

To maximize the advantages of slow solvent evaporation, we proposed an idea of sonication, followed by magnetic stirring at R.T. for emulsions. It is hypothesized that initial sonication process should be able to make stable emulsion at <39.6 °C and the level of evaporation can be simply controlled by adjusting stirring time. For this purpose, O/W emulsion was prepared by 5 min sonication both at R.T. and in iced water, and magnetically stir incubated at R.T. (at 700 rpm) for various time intervals (0, 2, 4, and 8 hrs). SEM data in Fig. S6 show that enough incubation of sonicated emulsion is important parameter to make spherical particles. Sonicated emulsion without incubation resulted in the formation of non-spherical morphology, however evolved into spherical shape with the increase of incubation time. In addition, longer incubation had a tendency to form bigger pores. Similarly, iced water incubation samples exhibited non-spherical morphology and further incubation had an increase in the population of spherical MPs as shown in Fig. S7. Thus, it is assumed that incubation time plays a critical role in determining emulsion stability, morphology of MPs, and size of pores. It is important to remember that while MP morphology deviates from the sphere, as far as non-spherical particles have pores, they can still be used for encapsulating drugs.

**Figure 6:**
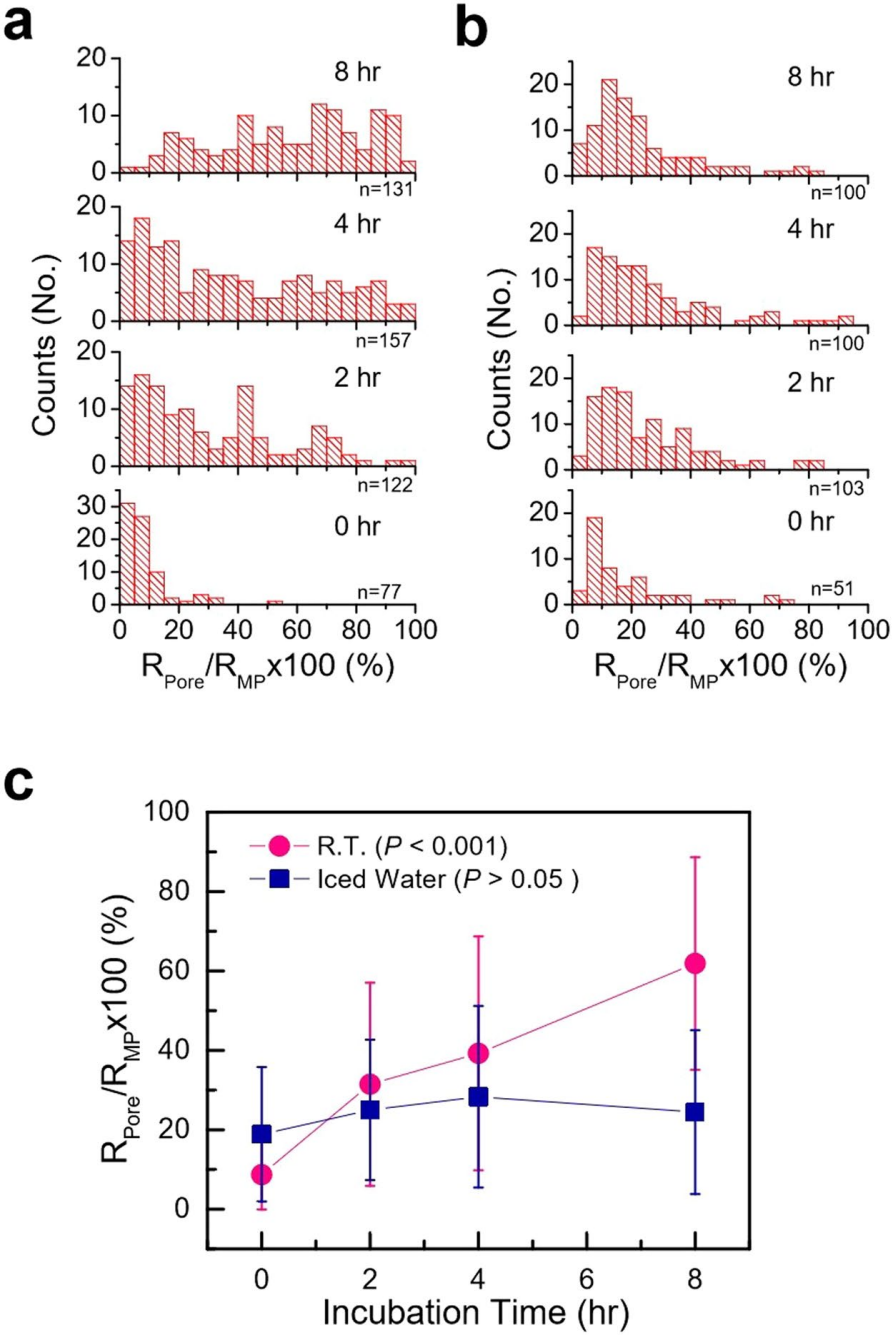
Histogram of pore to MP size ratio for MPs prepared by stir incubation. MPs were fabricated by 5-min sonication at (**a**) R.T. and (**b**) iced water conditions, followed by magnetic stirring for different time intervals, and their corresponding profile (**c**).

To further evaluate the effects of sample preparation conditions on the pore size of MPs, the relative size of pores and MPs was determined by analyzing SEM images (representative images were selectively shown in Figs S6 and S7). The data shows that the relative pore size of R.T. sonicated MPs tends to increase with incubation time in contrast to no significant change for iced sonicated MPs (R.T. MPs: ANOVA, *P* < 0.001, iced water MPs: ANOVA, *P* > 0.05; see histogram of R.T., Fig. 6(a), and iced water samples, 6(b), and 6(c) for analysis plots). This difference in pore size change can be explained by the different sample temperatures. That is, initial high temperature coupled with incubation at R.T. account for higher evaporation of solvents, leading to the formation of time-dependent pore size increase. In contrast to R.T. incubation, low sample temperature of iced water incubation samples can suppress temperature increase and slower the evaporation of solvents during sonication. This explains why no significant level of pore size increase was not observed from iced water samples. All these experiments provided the fundamental understanding of the evaporation rate of solvent during the MPs fabrication process, and were later can be used as the baseline for finalizing the protocol for fabricating MPs (30 min initial sonication, followed by stirring at R.T. for 10 min, and then 30 min additional sonication) with optimum quality control over morphology and their pores sizes.

While allowing for economic and efficient fabrication of the concepted smart pH-sensing pored MPs, emulsification by sonication presents its own technical challenges for controlling the quality of MPs. Microemulsion formulation (i.e., types/concentrations of polymers, solvents, and surfactants) and preparation process (i.e., temperature, pressure, viscosity, and dispersion conditions/methods) have been reported to significantly affect the characteristics of MPs, as also implied in Figs 5 and 6. In this study, the effect of emulsion temperature during sonication was tested under the hypothesis that control over solvent evaporation rate plays a critical role in forming macropores on MPs. This accounts for a strong correlation between pore size and the generation of heat due to sonication. However, mechanical agitation itself can also induce evaporation of solvents even at much lower temperatures than their boiling points. Thus we believe that sonication-induced solvent evaporation at the initial stage of emulsification process might increase difficulty in controlling a narrow pore size distribution, which explains relatively broad pore size distribution of MPs in this work. As a result, much efforts need to be put into careful selection of materials as well as precise control of emulsification conditions to yield uniform and better controlled pores with high yield.

Although our main research has been focused on fabrication of MPs made of anionic copolymers for intestine drug delivery, the use of sonication method was demonstrated to work for the synthesis of pored MPs using cationic copolymers (Eudragit EPO; poly(butyl methacrylate-co-(2-dimethylaminoethyl) methacrylate-co-methyl methacrylate)) for application in stomach-targeted drug delivery (see Fig. S8, Supporting Information)^52^. Further research will build upon these findings, which support the generality of the sonication method for the fabrication of pH responsive pored MPs, applicable to diverse materials with simple modifications in emulsion parameters.

In addition, it should be noted that the development of a process to form closed-pored MPs increased the feasibility of MP-based delivery systems by protecting functional ingredients inside MPs against the harsh environment conditions. The pores of MPs made of anionic polymers remained intact (closed) at gastric acidity, but opened at neutral intestinal pH, which was further confirmed by monitoring release behavior of functional ingredients (FNPs and SRB) from MPs. As a result, lactase-encapsulating MPs were effective in protecting lactase at low pH, leading to >15 times higher lactase activity than that of a commercial tablet formulation under simulated GI tract environment. Importantly, drug loading and drug release can be affected by pore size of MPs, drug concentration, and drug size. As compared in Fig. 3, small molecules (SRB) showed more rapid and efficient release behavior compared to large model drug (FNPs), indicating the importance of drug size in release behavior. This, in part, can be explained by the smaller opening of MP pores (0.85 ± 0.55 µm) in simulated intestinal fluid, i.e. release medium, compared to original MP pores (1.5 ± 0.7 µm) (see Figs 1c(ii) and 2c(iv)), which probably can result in more significant release resistance to 100 nm sized FNPs compared to SRB. Although the influence of pore size on drug loading/release characteristics was not investigated in this work, we expect that the decrease in the size of pores on MPs would increase resistance to drug loading, resulting in low encapsulation efficiency, and decrease the release rate of encapsulated drugs through their pores.

In this work, pH-responsive behavior of pored MPs was examined using simulated GI fluid without enzymes to eliminate interference between GI enzymes (pepsin for gastric fluid and pancreatin for intestinal fluid) and ONPG assay in determining lactase enzyme activity. However, it is imperative to validate the viability of oral drug delivery system in the presence of enzymes, considering its general application. To this end, we performed SEM analysis to characterize MPs with self-controlled macroscopic pores in response to simulated GI fluids with enzymes, and observed a similar behavior to the one observed from the simulated GI fluids without enzymes. That is, MPs maintained closed-pore state in simulated gastric fluid with pepsin (Fig. S9a, Supporting Information) and opened their pores in simulated intestinal fluid with pancreatin (Fig. S9b Supporting information). This indicates that pore closing/opening response is driven mainly by environmental pH change and demonstrates the potential *in vivo* application of the proof-of-concept pored MPs. From a previous work by Ghaffarian *et al*., it was found that the GI enzyme-containing GI fluids do not induce further degradation of encapsulated antibodies compared to the simulated GI fluids without enzymes^53^. Hence, closure/reopening of pores in the simulated GI fluids and maintenance of intact structure of lactase in response to pH change, as proven in this study, clearly indicate the potential of pored MPs for oral drug delivery system. Although not dealt with here, it should be noted that an important consideration in future *in vivo* studies includes the effects of non-specific and specific surface functionalization of pored MPs on mucoadhesion and target specificity, respectively.

**Figure 7:**
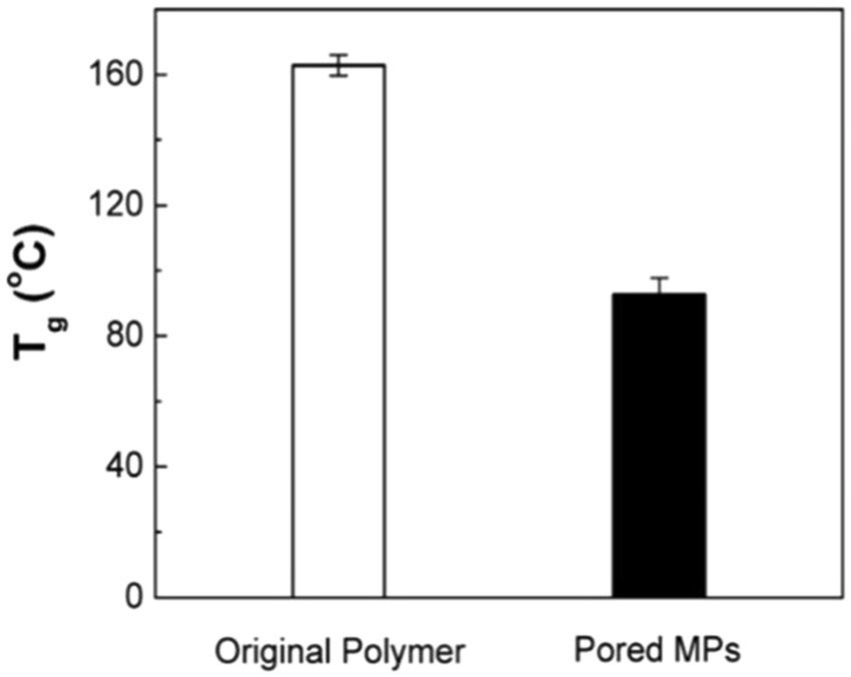
DSC analysis of original polymer and pored MPs (*n* = 3, mean ± SD).

The essence of pore closure is associated with intra-mass transfer in polymeric MPs without damaging the hollow interior. While detailed mechanism behind the pore closure is not fully understood, major reasons are probably related to the decrease of glass transition temperature (T_g_) and freeze-drying process. As shown in Figs S10 and S11 (Supporting Information), FTIR spectra and ^1^H-NMR analysis confirm the presence of Tween 20 into MPs post fabrication. Incorporation of Tween 20 into MPs lower their T_g_ due to its plasticizing effect and result in increased flexibility among polymer chains (see Fig. 7 for T_g_ measurements)^54^. Multiple studies have been reported, which utilize increased polymer chains mobility to seal the pores of size ranging from hundreds of nanometers to several micrometers^47, 55, 56^. However, pore closure by raising temperature above T_g_ of the microencapsulation might have limited range of application due to major concerns regarding stability of thermosensitive biopharmaceuticals such as vaccines. On the other hand, in our pore closure technique, when MPs are subjected to freeze-drying process, the removal of aqueous core may lead to the increase of hydrophobic interactions among polymers. Therefore, we hypothesize that lowering of T_g_ and freeze drying process facilitated the migration of polymer chains within MPs, which eventually led to sealing of pores. Although polymer and dehydration method are different, pore closure on polymer film has been previously reported to be related to polymer chain flexibility and inter-polymeric interaction^57^. In addition, recyclability of the MPs- defined as the ability to obtain the closed pored state post freeze-drying after being repeatedly subjected to pore opening/closing process, was confirmed for up to 10 freeze-drying cycle.

As such, our proof-of-concept pored MPs were proven to provide a solution to the maintenance of the high drug loading efficiency and the increased control over pH-dependent stabilization/release behavior. Considering that major technical challenges to oral drug delivery systems include stability of drugs in the harsh environment of GI tract and target-specific release in the intestine, our MPs with a pH-responsive macropore represent a potential alternative to conventional delivery systems. This was enabled by designing a smart architecture equipped with pH-sensing functionality. Lastly, to achieve the ultimate goal of universal platform for oral drug delivery, we also recognize that the posed technical challenges are the increase of pore closure efficiency, and the surface functionalization to enhance mucoadhesion and bioavailability.

## Conclusion

The hallmarks of the proposed oral drug delivery system are (i) a hybrid structure combing the advantages of both solid microparticles and hollow microcapsules (ii) a pH-responsive macropore, whose opening/closing is dependent on the environmental pH change. The presence of macropores allows easy solvent-free ingredient loading into hollow inner core of the systems, which has been long desired in oral drugs and vaccine delivery research. The principle of the protection and release of ingredients in the MPs depends on the opening-closing behavior of the pores. The closed-pored MPs at unfavorable pH protects functional ingredients by isolating the interior from the surrounding environment, and pore opening at favorable pH facilitates the release of the encapsulated substances. The facile sonication method was found to be applicable for fabricating MPs from Eudragit EPO polymer, validating the compatibility with other materials as well. Therefore, MPs based on tailored materials with pH-responsive pores for specific applications, can make a significant contribution to food science, drug/vaccine delivery, and human/animal health sciences.

## Electronic supplementary material

Supporting Information
