# Supplementary material for: Smart Microparticles with a pH-responsive Macropore for Targeted Oral Drug Delivery
Source: Sci Rep. 2017 Jun 8;7:3059. doi: 10.1038/s41598-017-03259-x (PMC5465058; doi:10.1038/s41598-017-03259-x)
Supplement: Supplementary file 1 — Supporting Information [file 41598_2017_3259_MOESM1_ESM.pdf]

Smart Microparticles with a pH-responsive Macropore for Targeted Oral Drug Delivery

Ankit Kumar, Carlo Montemagno*, and Hyo-Jick Choi*

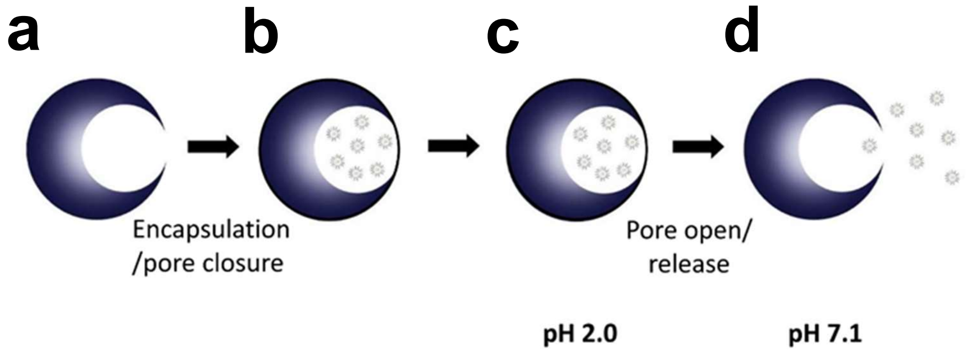

Figure S1. Schematic representation of proposed hollow microparticles (MPs) with a macropore. (**a**) pored MPs, (**b**) encapsulation of ingredients and pore closure, (**c**) protection and (**d**) release of functional ingredients under (**c**) acidic/(**d**) neutral/basic environments. Particle size variation is not taken into account.

Step	Shelf (°C)	Ramp (min)	Hold (min)	Vacuum (mTorr)
Primary drying				
1	–40	0	30	1000
2	–55	60	1	100
3	–55	0	2000	100
Secondary drying				
4	30	180	120	300

Figure S2. Recipe for freeze drying.

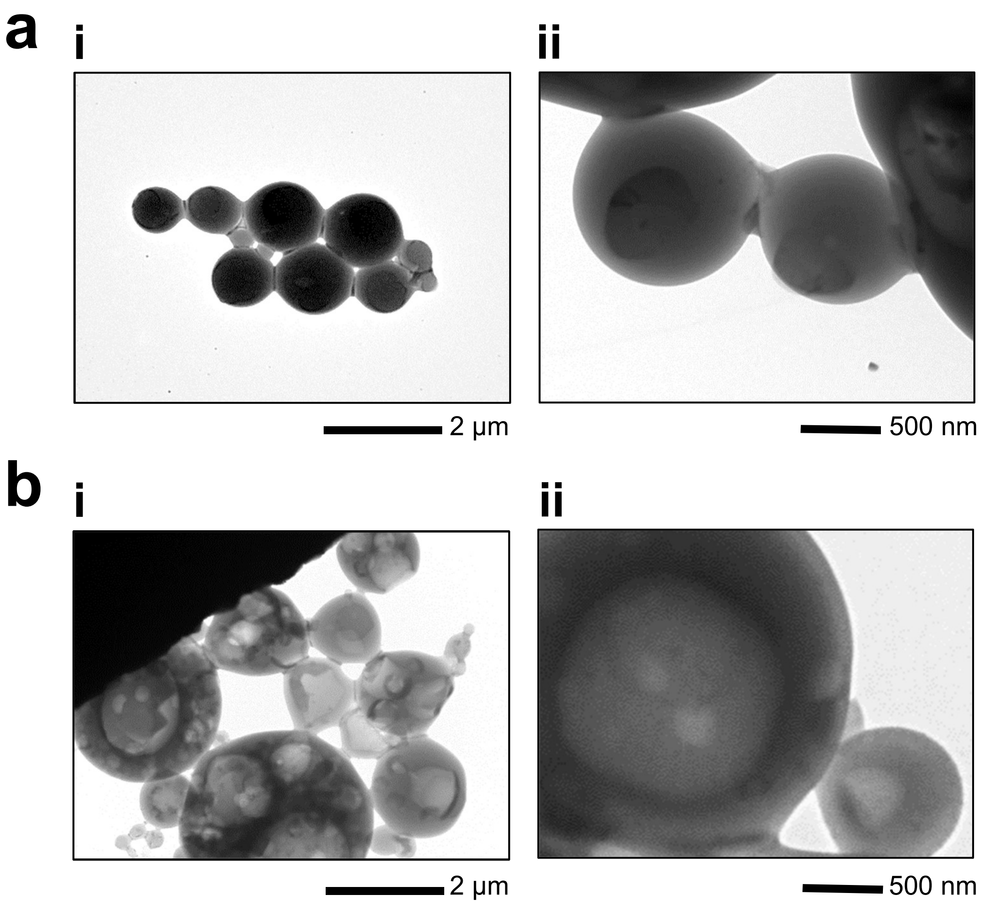

Figure S3. Pore closure and opening in response to pH. TEM images of MPs after being subjected to simulated GI tract conditions ((**a**) 2-hr incubation at pH 2.0, followed by (**b**) 4-hr incubation at pH 7.1 at 37°C, i: low mag. and ii: high mag.).

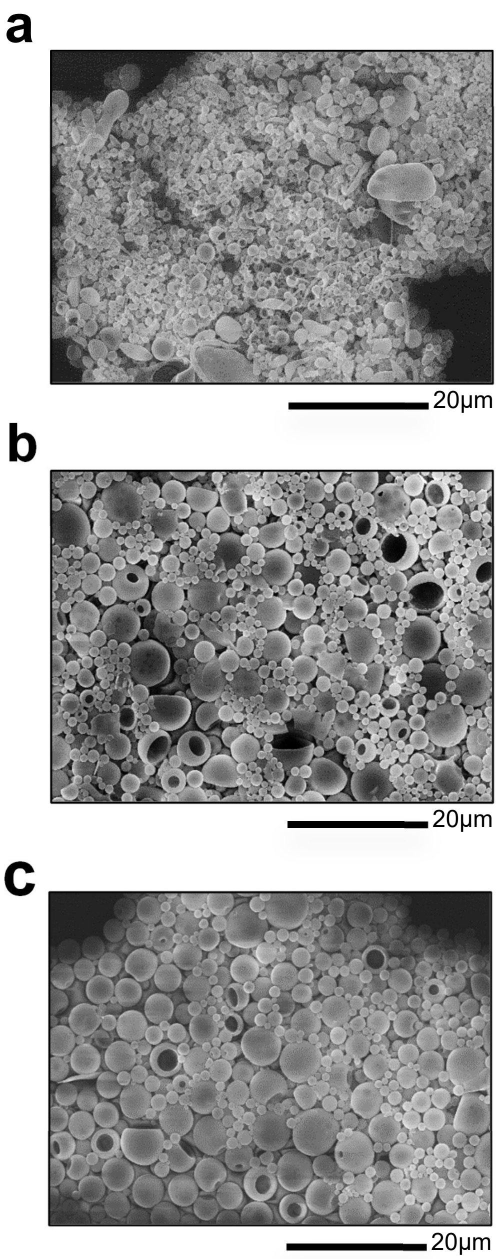

Figure S4. SEM images of MPs prepared by room temperature (R.T.) sonication conditions deployed for different time intervals: (**a**) 5 min, (**b**) 30 min, and (**c**) 60 min sonication.

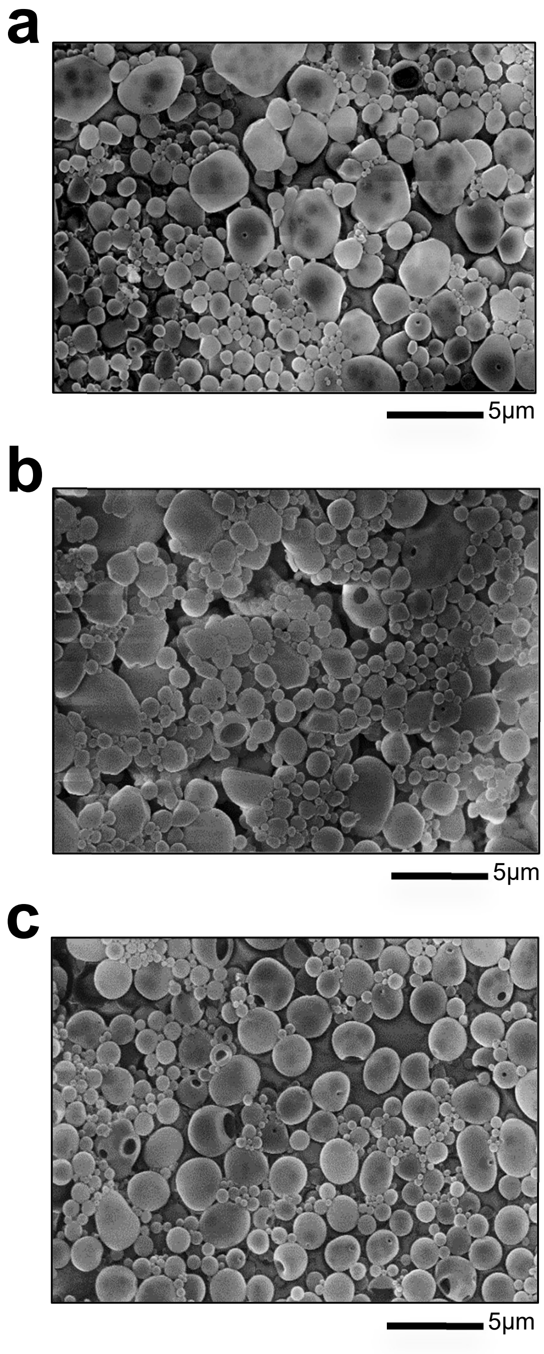

Figure S5. SEM images of MPs prepared in iced water sonication (sample temperature controlled by iced water surrounding it) for different sonication conditions deployed for different time intervals: (**a**) 5 min, (**b**) 30 min, and (**c**) 60 min sonication.

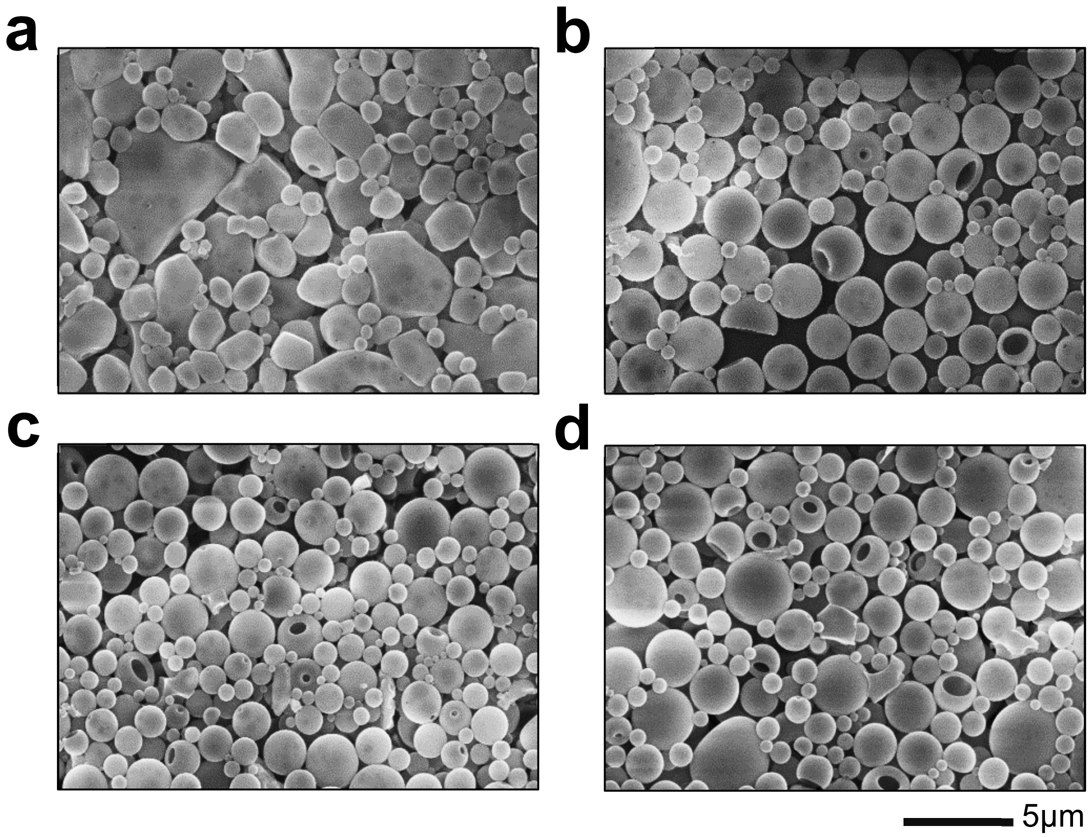

Figure S6. SEM images of pored MPs prepared by 5 min R.T. sonication, followed by stir incubation for different time intervals: (**a**) 0 hr, (**b**) 2 hrs, (**c**) 4 hrs, and (**d**) 8 hrs.

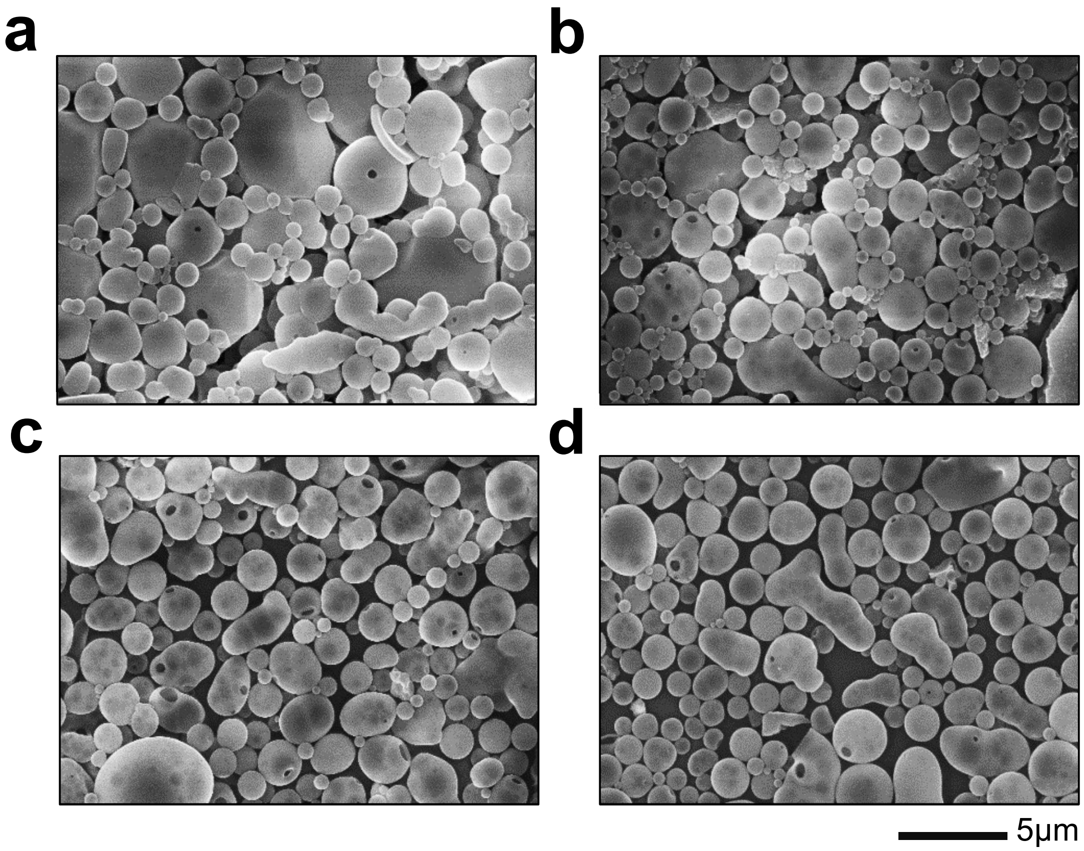

Figure S7. SEM images of pored MPs prepared by 5 min iced water sonication, followed by stir incubation for different time intervals: (**a**) 0 hr, (**b**) 2 hrs, (**c**) 4 hrs, and (**d**) 8 hrs.

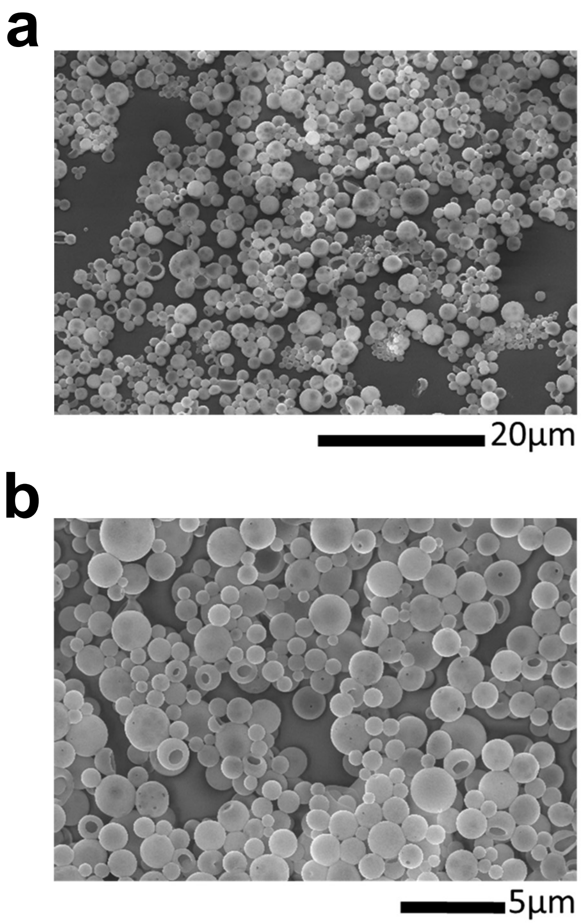

Figure S8. SEM images of MPs with a macropore synthesized from Eudragit EPO, a cationic copolymer. (**a**) Low mag. and (**b**) high mag.

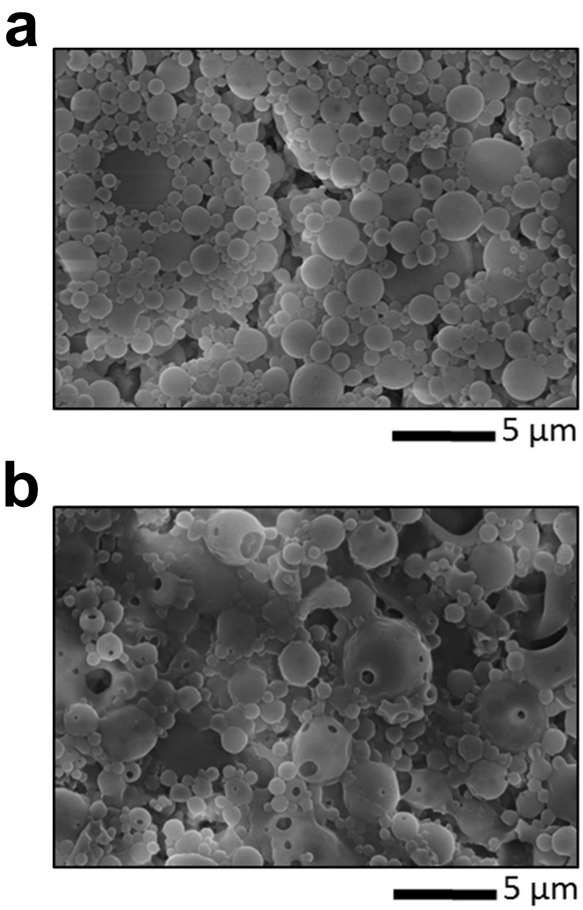

Figure S9. SEM images of MPs after being subjected to **(a)** 2-hr incubation in simulated gastric fluid with pepsin (at pH 2.0 and 37°C) and **(b)** simulated GI tract conditions (2-hr incubation in simulated gastric fluid, followed by 4-hr incubation in simulated intestinal fluid with pancreatin at pH 7.1 and 37°C).

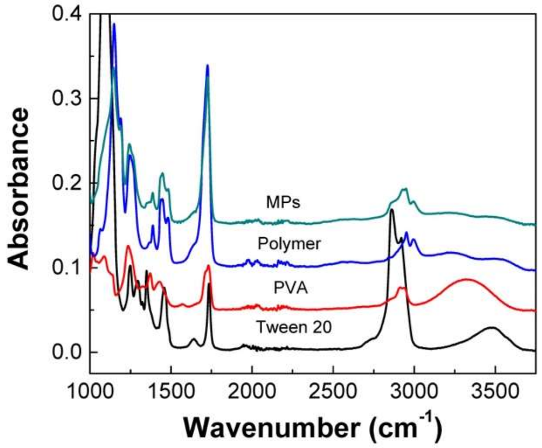

Figure S10. FTIR spectra of MPs, original polymer, PVA, and Tween 20. MPs exhibited bands at 2860 cm^{-1} and 2920 cm^{-1}, which is consistent with characteristic peaks of Tween 20 and Tween 20/PVA, respectively. The presence of characteristic Tween 20 peak on MPs at 2860 cm^{-1} indicates that Tween 20 has been incorporated into MPs. However, the characteristic peak at 2920 cm^{-1} can correspond to Tween 20 or PVA.

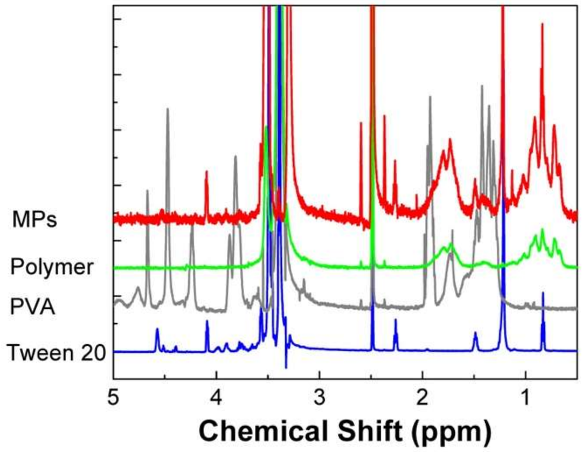

Figure S11. ^{1}H NMR analysis of MPs, original polymer, PVA, and Tween 20. From the comparison of ^{1}H-NMR spectra of pored MPs with original polymer, Tween 20, and PVA in DMSO solvent, it was confirmed that only Tween 20 was incorporated.
